# Aggregation, impaired degradation and immunization targeting of amyloid-beta dimers in Alzheimer’s disease: a stochastic modelling approach

**DOI:** 10.1186/1750-1326-7-32

**Published:** 2012-07-02

**Authors:** Carole J Proctor, Ilse Sanet Pienaar, Joanna L Elson, Thomas BL Kirkwood

**Affiliations:** 1 Institute for Ageing and Health, Newcastle University, Campus for Ageing and Vitality, Newcastle upon Tyne, NE4 5PL, United Kingdom; 2 Institute of Genetic Medicine, Newcastle University, Central Parkway, Newcastle upon Tyne, NE1 3BZ, United Kingdom

**Keywords:** Alzheimer’s disease, Amyloid-beta, Dimers, Down’s syndrome, Intervention, Immunotherapy, Mathematical model, Protein aggregation, Stochastic simulation

## Abstract

**Background:**

Alzheimer’s disease (AD) is the most frequently diagnosed neurodegenerative disorder affecting humans, with advanced age being the most prominent risk factor for developing AD. Despite intense research efforts aimed at elucidating the precise molecular underpinnings of AD, a definitive answer is still lacking. In recent years, consensus has grown that dimerisation of the polypeptide amyloid-beta (Aß), particularly Aß_42_, plays a crucial role in the neuropathology that characterise AD-affected post-mortem brains, including the large-scale accumulation of fibrils, also referred to as senile plaques. This has led to the realistic hope that targeting Aß_42_ immunotherapeutically could drastically reduce plaque burden in the ageing brain, thus delaying AD onset or symptom progression. Stochastic modelling is a useful tool for increasing understanding of the processes underlying complex systems-affecting disorders such as AD, providing a rapid and inexpensive strategy for testing putative new therapies. In light of the tool’s utility, we developed computer simulation models to examine Aß_42_ turnover and its aggregation in detail and to test the effect of immunization against Aß dimers.

**Results:**

Our model demonstrates for the first time that even a slight decrease in the clearance rate of Aß_42_ monomers is sufficient to increase the chance of dimers forming, which could act as instigators of protofibril and fibril formation, resulting in increased plaque levels. As the process is slow and levels of Aβ are normally low, stochastic effects are important. Our model predicts that reducing the rate of dimerisation leads to a significant reduction in plaque levels and delays onset of plaque formation. The model was used to test the effect of an antibody mediated immunological response. Our results showed that plaque levels were reduced compared to conditions where antibodies are not present.

**Conclusion:**

Our model supports the current thinking that levels of dimers are important in initiating the aggregation process. Although substantial knowledge exists regarding the process, no therapeutic intervention is on offer that reliably decreases disease burden in AD patients. Computer modelling could serve as one of a number of tools to examine both the validity of reliable biomarkers and aid the discovery of successful intervention strategies.

## Background

Alzheimer’s disease (AD) is characterised by cholinergic neuron loss, synaptic dysfunction and the accumulation of protein aggregates in specific regions of the brain [1]. The main brain regions affected are the cortex and hippocampus leading to clinical symptoms including the inability to form new memories and altered personality [2,3]. Although the underlying molecular causes remain unresolved, two functionally different proteins are involved in the aggregation process: amyloid-beta (Aβ), which forms extracellular plaques, and the microtubule binding protein tau, the main component of neurofibrillary tangles, with the relative contribution of each to the manifestation of disease symptoms remaining a matter of considerable controversy [4-6]. The order of events in the disease process is also unclear. Aβ is produced by the sequential processing of the amyloid precursor protein (APP), by means of a catalytic process involving the β- and γ-secretase enzymes. The Aβ peptide contains 36–43 amino acids with the actual length affecting its properties. For example, the isoform Aβ_40_ is normally secreted and less cytotoxic than Aβ_42,_ which has a strong tendency to aggregate in the brain and associates with AD [7]. Levels of Aβ_40_ and Aβ_42_ also increase in normal ageing [8] and have been shown to be elevated in the brains of Down’s syndrome patients [9]. An association has been made between the underlying genetic-molecular profile of AD and that of Down’s, with the detection of overexpressed *APP*, located on chromosome 21 [10]. Henceforth, elevated plasma concentrations of Aβ_40_ and Aβ_42_ were regarded as prominent risk factors for the onset of dementia in persons suffering from Down’s syndrome [11].

**Figure 1:**
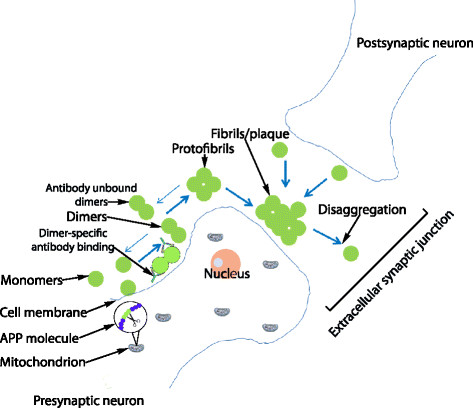
**Cartoon of Aβ aggregation.** Aβ monomers form dimers which then go on to form protofibrils and fibrils/plaques. Blue arrows indicate the aggregation/disaggregation process. Black arrows are used to make labelling clearer. Note the possibility that APP and Aβ may accumulate in mitochondria is also shown.

The aggregation process begins with the formation of soluble dimers and larger oligomers (e.g. tetramers and octamers) which interact to form protofibrils [12]. Protofibrils can be defined as transient structures which are observed during the lag phase of amyloid growth. They may disaggregate but can also grow by the addition of soluble Aβ, to finally form insoluble fibrils and plaques [13] (Figure 1). The issue concerning the relative contribution made by monomers, dimers, oligomers, protofibrils, fibrils and plaques to cell toxicity and the eventual onset of AD symptoms has elicited considerable debate [14]. It has been suggested that dimers, oligomers and protofibrils are more toxic than plaques [15]. In this regard, it was recently suggested that Aβ dimers are particularly toxic based on evidence that they are capable of blocking Long-Term Potentiation (LTP [16]. In this regard, Shankar and colleagues found that soluble forms of Aβ correlated best with AD compared to other forms of dementia [16]. In another study, Rowan and co-workers showed that human cerebrospinal fluid (CSF) containing Aβ dimers inhibited LTP *in vivo* but that the inhibition of LTP did not occur if CSF samples contained only monomers [17]. This study also showed that systemic passive immunization prevented LTP inhibition by dimers. Clinical evidence for the role of dimers in the disease state comes from the Cognitive Function and Ageing Studies (CFAS) which show that the sodium dodecyl sulfate (SDS)-stable soluble Aβ dimers found in the temporal and frontal cortex are strongly associated with AD [18]. In addition, it has been shown that dimers rapidly form protofibrils giving further support to the idea that dimers are the main building blocks of Aβ assemblies [19]. The growing recognition of the importance of dimers in the aggregation process has led to the development and testing of antibodies directed against dimers. For example, a monoclonal antibody that specifically recognises pathogenic forms of dimers has recently been shown to neutralise synaptotoxic effects in rats injected with Aβ dimers and monomers [20]. Aβ monomers are usually cleared from the cell by various mechanisms to prevent protein aggregation. Clearance mechanisms include degradation by proteases such as neprilysin and insulin-degrading enzyme (IDE) and transport away from the brain via the vascular system [21]. However, amyloid plaques do accumulate in the brain with age and are particularly associated with AD, suggesting impairment in the homeostatic control of formation versus clearance of Aβ. Mawuenyega and colleagues [22] measured the production and clearance of Aβ (both Aβ_40_ and Aβ_42_) in the CSF of AD patients compared to age-matched, neurologically-intact controls. Their study showed that the production rate of Aβ is the same in AD as in controls, but detected a slower degradation rate in AD. This suggests that Aβ degradation kinetics may be impaired in AD. The mechanisms underlying this alteration remain unknown. However, protein levels of neprilysin, a neutral endopeptidase, and one of the major Aβ-degrading enzymes located in the brain, have been shown to decline with age [23,24]. This may be attributable to oxidative damage since an increase in oxidatively-damaged neprilysin was found in AD subjects compared to normal controls [25]. Other studies have shown that the activity of neprilsyin increases with age in the normal brain [26] and in AD [27], although the latter study showed decreased neprilysin levels with age in AD subjects. The potential for targeting Aβ-degrading enzymes has been recently reviewed [28] and motivated the inclusion of neprilysin in our model.

The molecular mechanisms involved in AD are therefore complex and as yet we lack clear understanding of which part of the aggregation process would provide the best target for stopping or slowing down AD-related disease processes. In such complex scenarios where it has proved difficult to disentangle the contributing disease processes, mathematical modelling is increasingly being used to complement experimental approaches [29-32]. For instance, modelling can be used to integrate multiple mechanisms, refine complex hypotheses, make testable predictions and explore *in silico* the possible effectiveness of interventions. We have used dynamic modelling which means that the model can be used to examine mechanisms leading to the disease state. This has an advantage over experimental approaches which are often limited to the end time-point of the disease. Many biological processes are inherently stochastic. For example, protein aggregation involves random molecular interactions to initiate the formation of pathogenic structures [33]. Therefore stochastic computer models can be essential for examining the plausibility of alternative mechanisms. To this end, we built a stochastic model of turnover of Aβ_42_ (hereafter referred to as Aβ) to investigate the effects of the degradation rate on Aβ levels and specifically to discover whether a slower degradation rate is itself sufficient to explain the increased level of soluble Aβ and senile plaques seen in AD-affected brains, a molecular phenomenon which is strongly believed to underlie disruption of memory function [16,18,34]. The potential clinical importance associated with an intervention strategy to lower soluble Aβ levels was demonstrated by Busche and others [35], who recently provided evidence in support of the concept that soluble Aβ induces neuronal dysfunction. This was experimentally revealed by direct application of soluble Aβ in wild-type mice, who then displayed pathological neuronal hyperactivity within the hippocampus region. This deficit was rescued through acute treatment with a γ-secretase inhibitor, which reduced soluble Aβ levels. Importantly, the authors observed that the selective increase in neuronal hyperactivity preceded the formation of plaques, to suggest that soluble species of Aβ may underlie this functional neuronal impairment. We then extended this model to include details of Aβ aggregation, running simulations over long periods of simulated time (up to 100 years) in order to examine the aggregation process in human ageing. We used the model to compare the plausibility of different candidate mechanisms and, by varying key parameters in the model, to identify which processes are most sensitive to modification and might therefore be best to target in order to reduce the burden of plaques.

## Results

### Model 1: Aβ turnover

#### A lower degradation rate of Aβ has only a small effect on soluble levels of the protein but increases the probability that monomers will exceed a critical threshold

**Figure 2:**
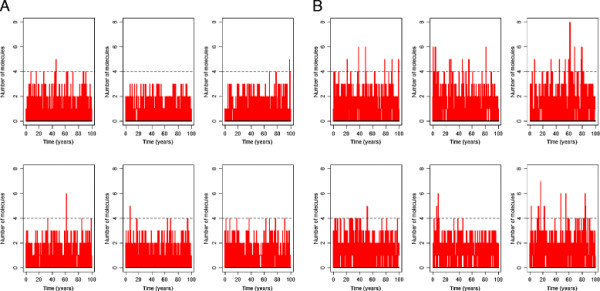
**Simulation output for model of Aβ turnover.** The graph shows the level of Aβ versus time over a 100 year period in 6 typical runs. A *k*_*degAbeta*_ = 2.1 e-5 s^-1^ B *k*_*degAbeta*_ = 1.5 e-5 s^-1^. The horizontal dashed lines correspond to the assumed threshold required for dimerisation to occur.

**Figure 3:**
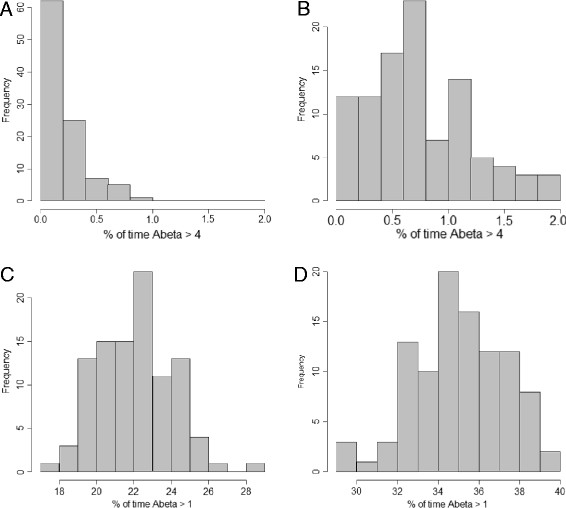
**Histograms showing percentage of times that Aβ exceeds threshold for normal and AD degradation rates. A:** threshold = 4, *k*_*degAbeta*_ = 2.1 e-5 s^-1^; **B:** threshold = 4, *k*_*degAbeta*_ = 1.5 e-5 s^-1^; **C:** threshold = 1, *k*_*degAbeta*_ = 2.1 e-5 s^-1^; **D:** threshold = 1, *k*_*degAbeta*_ = 1.5 e-5 s^-1^. In each case, 100 simulations were carried out and 500 time-points were recorded.

This model showed that the difference in degradation rate has only a small effect on soluble Aβ levels. Aβ does not accumulate with time but just has a slightly higher level in AD compared to normal (Figure 2). However, although levels of soluble Aβ do not accumulate with time, the slightly higher levels of Aβ throughout life increase the chance of aggregation. Due to stochastic effects, Aβ levels occasionally increase above normal basal levels for short periods of time and it may be possible that dimers are formed during theses phases as shown by the intermittent peaks in Figure 2. The first step in the aggregation process is the formation of dimers, and since the probability of dimer formation depends on the number of Aβ molecules, we assume that dimer formation is unlikely to occur unless the number of Aβ molecules exceeds a threshold. For example we could assume that the threshold is four and then count the number of times that Aβ takes values that are greater than four in each simulation. The model predicts that Aβ exceeds the threshold for a larger percentage of times when the degradation rate is lower for either a threshold of four or if the threshold is lowered to one (Figure 3). In particular, when the threshold is one, there is no overlap between the percentage of times that this threshold is reached or exceeded between the normal and AD degradation rates. Since we would expect plaques to be rare when Aβ degradation rates are normal, our model suggests that a threshold of one is biologically plausible.

**Figure 4:**
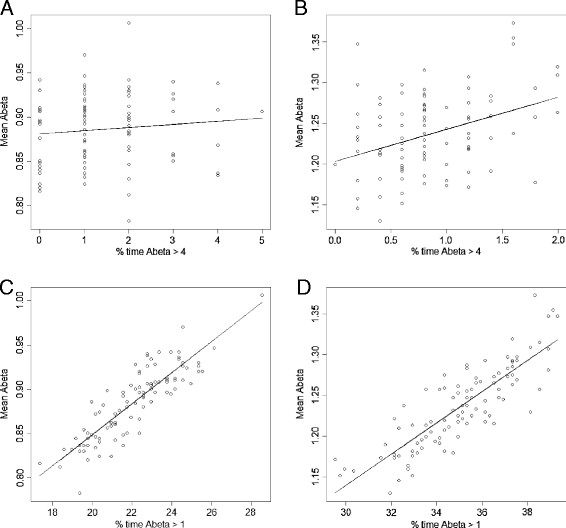
**Linear regression analysis for mean Aβ levels versus percentage of times that Aβ exceeds two different thresholds for normal and AD degradation rates. A:** threshold = 4, *k*_*degAbeta*_ = 2.1 e-5 s^-1^; **B:** threshold = 4, *k*_*degAbeta*_ = 1.5 e-5 s^-1^; **C:** threshold = 1, *k*_*degAbeta*_ = 2.1 e-5 s^-1^; **D:** threshold = 1, *k*_*degAbeta*_ = 1.5 e-5 s^-1^. In each case, 100 simulations were carried out and 500 time-points were recorded.

Linear regression analysis showed that the slope of the line was not significantly different from zero for normal Aβ degradation when the threshold is four but it was highly significant (*p* = 0.00019) for the AD degradation rate (Figure 4A, B). There was no significant correlation between mean Aβ levels and the percentage of times that Aβ levels exceeded a threshold of four for normal degradation rate (Pearson’s r = 0.10) or AD degradation rate (Pearson’s r = 0.36). When the threshold was reduced to one, the slope of the line was highly significantly different from zero for both degradation rates (*p* < 0.0001) and there was a strong positive correlation between mean Aβ levels and the percentage of times that the threshold was exceeded (normal degradation rate, Pearson’s r = 0.87; AD degradation rate, Pearson’s r = 0.84) (Figure 4C, D). The mean Aβ level was significantly higher for the AD degradation rate compared to the normal degradation, as seen by the lack of overlap in the range of values (compare y-axis values in Figure 4A and B or Figure 4C and D). The percentage of times that Aβ exceeded any given threshold was also significantly higher for the AD degradation rate (compare x-axis values in Figure 4A and B or Figure 4C and D). Therefore the lower degradation rate seen in AD may account for the aggregation of Aβ in disease progression. To examine this possibility we extended the model to include steps for Aβ aggregation.

### Model 2: Aβ turnover and aggregation

Model 1 was extended by adding reactions for the formation of dimers and aggregates, assuming that these processes were reversible. We defined a plaque to be an aggregate of size greater than 30 monomers as this was the size at which according to the model output, an aggregate had a very low probability of disaggregating completely. Full details of our terminology are given in the Methods section.

#### Decreased clearance of soluble Aβ leads to plaque formation

**Figure 5:**
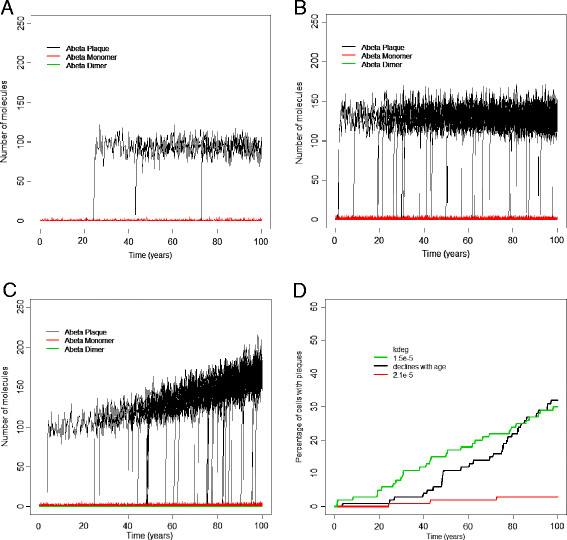
**Output from 100 runs for different Aβ degradation rates. A-C** Plots of plaques versus age from 100 simulated cells. A *k*_*deg*_ = 2.1e-5, B *k*_*deg*_ =1.5e-5, C *k*_*deg*_ decreases linearly with age, D Plot of percentage of simulated cells associated with plaques versus age for each of the three Aβ degradation rates shown in **A-C.**

The model predicts that very few plaques are formed when Aβ clearance is efficient (Figure 5A). If we assume that the degradation of Aβ is lower throughout life then the model predicts that plaques form at any age, with just as many forming before the age of 60 as in later years (Figure 5B), and there is a linear increase in the number of cells associated with plaques with age (Figure 5D, green curve). However, if we assume that Aβ clearance gradually declines with age, the model predicts that the majority of plaques form after age 60 (Figure 5C) and there is an exponential increase in the number of cells associated with plaques with age (Figure 5D, black curve). Therefore, it is much more realistic to assume that the rate of Aβ clearance declines with age in patients with a higher risk of AD, rather than assuming impaired degradation from birth. It should be noted that the graphs in Figure 5D show the percentage of cells associated with plaques as a function of time and that the actual size of plaque within each cell reach a maximum size (see Figure 5C) and so the plaque burden associated with particular sets of neurons reaches a plateau rather continuing to increase in size.

We assumed that the rate of Aβ clearance declined with age in AD due to the decline in neprilysin protein levels over time. To see whether the model also predicted a change in neprilysin activity with age, we added a dummy variable to the model which counted each neprilysin-dependent Aβ degradation reaction. A plot of this dummy variable versus age is linear indicating that the reaction rate does not change with time (not shown). The reaction contains both neprilysin and Aβ as reactants and so a decline in neprilysin with time means that there must be an increase in the number of Aβ monomers in order that the reaction rate remains constant. Therefore our model predicts that the actual activity per neprilysin molecule gradually increases with time due to higher levels of monomers. However, despite the increase in neprilysin activity, the levels of monomers are higher so that there is a greater chance of dimers forming which may then initiate the aggregation process.

### Parameter scans for model 2

#### Dimerisation rate has the largest effect on the number of plaques formed and the age of onset of plaque formation

We carried out parameter scans for each individual parameter in Model 2 for the normal degradation rate, where we examined the effect on plaque formation. We considered the average lag time (time to when plaques first appear), plaque size and the percentage of cells associated with plaques at different ages. We summarize the parameters affecting each of these outcomes in Table 1. For each parameter scan we carried out 500 simulations and calculated the mean percentage of cells associated with plaques at different time-points.

**Table 1:** Effect of increasing parameter values on model outputs for normal Aβ degradation rate (Model 2)

**Parameter**	**Number of plaques (%)**	**Plaque size**	**Lag time**
*k*_*dimer*_	Increases to max of 100	No effect	Decreases
*k*_*dedimer*_	Decreases	No effect	Increases
*k*_*pf*_	Increases to max of ~50	No effect	No effect
*k*_*pg*_	Small effect	Increases	No effect
*k*_*pghalf*_	Small effect	No effect	Slight decrease for large values
*k*_*disagg*_	No effect	Decreases	Increase at high values
*k*_*degNep*_	Increases	Increases	Decreases

### Varying *k*_*dimer*_

**Figure 6:**
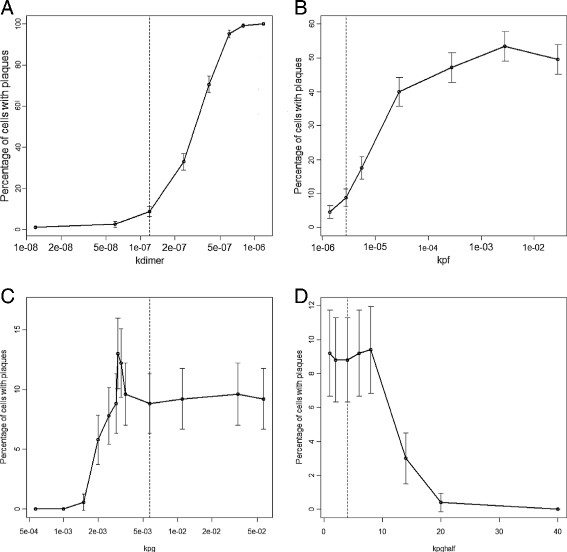
**Effect of varying parameter values on plaque formation. A***k*_*dimer*_, **B***k*_*pf*_, **C***k*_*pg*_, **D***k*_*pghalf*_. Each point shows the percentage number of cells associated with plaques at age 100 years from 500 simulations. The error bars show 95% confidence intervals for the percentages. The dashed vertical line indicates the default parameter value. In **B** the x-axis is plotted on a log scale as the parameter values were varied over four orders of magnitude. In **A** and **C** the x-axis is plotted on a log scale as the parameter values were not linearly spaced.

As the value of *k*_*dimer*_ increases, the percentage of cells associated with plaques increases as expected, since increasing *k*_*dimer*_ leads to more dimers and so an increased probability of plaque formation. However, there was no effect on plaque size since *k*_*dimer*_ only affects the initial step in plaque formation and not subsequent growth which depends on the pool of monomers rather than dimers. A plot of the percentage number of cells associated with plaques at age 100 years versus *k*_*dimer*_ is sigmoidal so that very low values of *k*_*dimer*_ lead to very low numbers of plaques, followed by an exponential increase and then a flattening off until eventually all cells are associated with plaques (Figure 6A). It is not realistic that all cells would be associated with plaques by age 100 years and so large values of *k*_*dimer*_ are not biologically plausible. The parameter *k*_*dimer*_ has a large effect on the lag time with a negative correlation between *k*_*dimer*_ and median lag time. Varying the parameter *k*_*dedimer*_ had the opposite effect to varying *k*_*dimer*_ (data not shown).

### Varying *k*_*pf*_

Increasing the value of *k*_*pf*_ leads to an increase in the percentage of cells associated with plaques, up to a maximum of about 50% (Figure 6B). Even increasing *k*_*pf*_ over four orders of magnitudes did not result in all cells being associated with plaques by age 100 years. This is because the rate of plaque formation is dependent on the pool of dimers and so can only increase if there are dimers available. Changing *k*_*pf*_ has no effect on the size of plaques or the lag time.

### Varying parameters for plaque growth

The parameters which affect plaque growth are: *k*_*pg*_ (which affects the maximum rate) and *k*_*pghalf*_ (which is the plaque size at which the growth rate is 50% of the maximum rate). The rate of growth also depends on the plaque size and the availability of Aβ monomers. The model predicts that increasing *k*_*pg*_ initially leads to an increase in the percentage of cells associated with plaques but reaches a maximum of just over 12% and then there is a sharp decline to about 10% which remains fairly constant with further increases of the parameter (Figure 6C). However, this behaviour is due to stochastic effects as only small numbers of cells developed an association with plaques, as can be seen by the 95% confidence interval (CI) error bars. Overall, the effect of increasing *k*_*pg*_ on the number of affected cells is small since if plaques are not formed in the first place, they cannot grow. As expected increasing the value of *k*_*pg*_ leads to larger plaques and has no effect on the lag time. Increasing the value of *k*_*pghalf*_ reduces the aggregation rate and so the number of plaques decreases, but there is no effect on plaque size.

### Varying *k*_*disagg*_

Increasing the rate of disaggregation *k*_*disagg*_ has no effect on the percentage of cells associated with plaques or the lag time. However, increasing this parameter has a large negative effect on plaque size. This is not surprising as disaggregation removes monomers from the plaques but is not involved in the actual formation of plaques.

### Varying rate of decline of neprilysin

**Figure 7:**
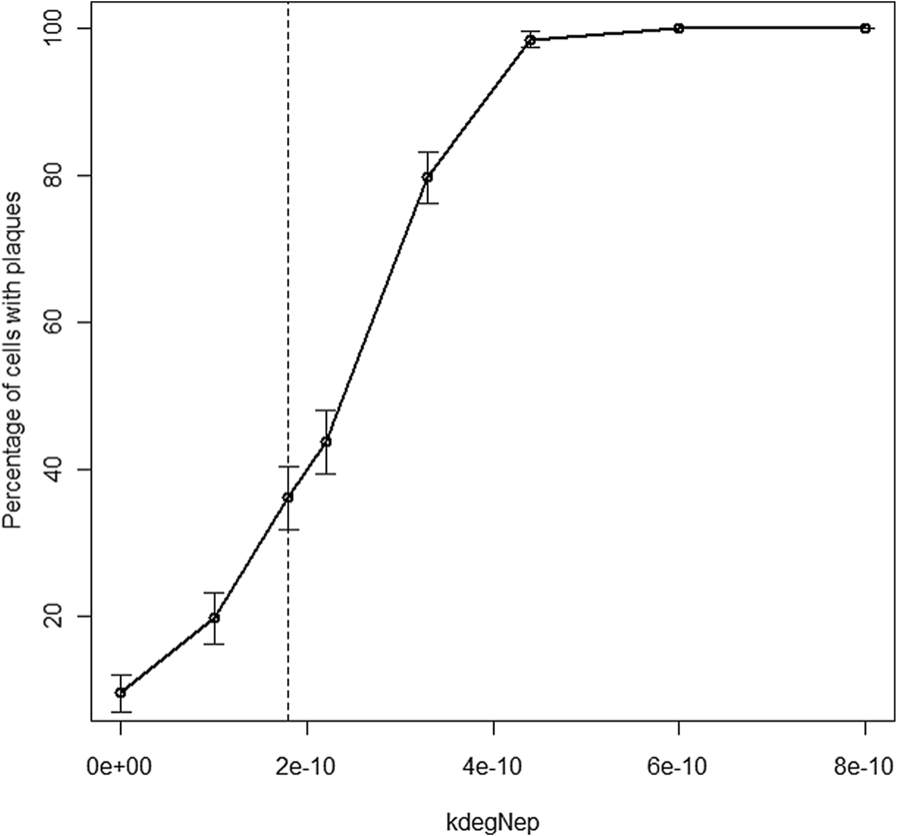
**Varying *k* **_** *degNep* **_** *.* ** Each point shows the percentage number of cells associated with plaques at age 100 years from 500 simulations. The error bars show 95% confidence intervals for the percentages. The dashed vertical line indicates the default parameter value.

Increasing the rate at which neprilysin declines leads to an increase in both the number and size of plaques and also decreased the lag time (Figure 7, Table 1). This is because the levels of neprilysin affect Aβ degradation so that if levels of neprilysin decline more rapidly, Aβ degradation is impaired at an earlier age.

**Table 2:** List of parameter values for Model 2

**Parameter**	**Description**	**Value**	**Comment**
*k*_*prod*_	Aβ production rate	1.86e-5 molecules s^-1^	Value from Mawuenyega at al. 2010 [22]
*k*_*deg*_	Aβ degradation rate	2.10e-5 s^-1^	Values from Mawuenyega et al. 2010 [22]
*k*_*dimer*_	Aβ dimerisation rate	1.2e-7 molecules^-1^ s^-1^	Chosen from global parameter scan
*k*_*dedimer*_	Dissociation rate of dimers	8.5e-6 s^-1^	Chosen from global parameter scan
*k*_*pf*_	Formation rate of plaques	2.8e-6 molecules^-1^ s^-1^	Value in range of 1 new aggregate per 5 days (Lomakin et al., 1997) [13]
*k*_*pg*_	Maximum rate of plaque growth	5.7e-3 s^-1^	Approx 5 monomers per min (Lomakin et al. 1997) [13]
*k*_*pghalf*_	Size of plaque at which growth rate is half of *k*_*pg*_	4.0 molecules	Chosen from global parameter scan
*k*_*disagg*_	Rate of plaque disaggregation	5.4e-5 s^-1^	Chosen from global parameter scan
*k*_*degNep*_	Neprilysin degradation rate	0.0 s^-1^	Normal rate (neprilysin remains constant with age)
		1.8e-10 s^-1^	Set so that neprilysin declines to about 700 by age 60 years.

### Addition of antibodies against Aβ dimers

#### Model predicts that early interventions have largest benefits, although late interventions are also beneficial

**Figure 8:**
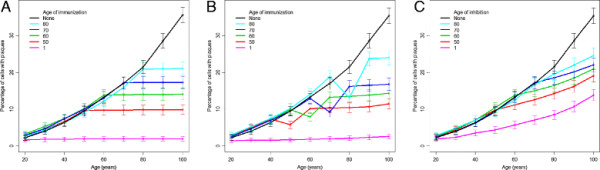
**Simulated interventions. A** Simulated vaccination with antibodies against Aβ dimers at different ages. **B** Simulated vaccination with antibodies against Aβ monomers at different ages. **C** Simulated inhibition of Aβ production by 25% at different ages. **A-C** Each point shows the percentage number of cells associated with plaques at age 100 years from 2000 simulations. The error bars show 95% confidence intervals for the percentages. Parameters used in **A:***k*_*binantiAbDim*_ = 1.0e-7s^-1^, *k*_*prodantiAb*_ = 1.0 e-7s^-1^; parameters used in **B:***k*_*binantiAbMon*_ = 1.0e-7s^-1^, *k*_*prodantiAb*_ = 1.0 e-5s^-1^.

To mimic the effect of adding antibodies against Aβ dimers we added to the model the molecular species named “antiAbDim”, which represents an antibody that binds to Aβ dimers [20,36]. We added two reactions: one for the binding of antiAbDim to AbDim, and another for the continued addition of antiAbDim. We assumed that initially the level of antiAbDim = 0, and that no production of antibodies takes place. In order to mimic the injection of antibodies at different ages, we added an SBML event structure to the model which set the level of antiAbDim to 1000 and the parameter for continued addition of antibody to 1.0e-7 s^-1^ at different time-points during the simulation. We simulated the effect of adding antibodies at age 50, 60, 70 and 80 years to examine at which age the model predicts antibody treatment would be most effective. We also simulated the addition of antibodies at age one year to examine whether the model would predict that vaccinating babies with Down’s syndrome would prevent the accumulation of plaques at early ages. Importantly, our model predicts that immunotherapy is much more beneficial if delivered at an early age while plaque levels are still low, but that immunization delivered even at later ages is still beneficial (Figure 8A). It is particularly noteworthy that immunization at one year of age completely prevents the accumulation of plaques with age and suggests a potential therapy to prevent dementia in Down’s syndrome affected patients.

### Addition of antibodies against Aβ monomers

#### Model predictions suggest that this intervention may be less effective than antibodies against Aβ dimers

Another therapeutic approach which is currently under consideration is the use of antibodies against Aβ monomers [[37], reviewed by[38]]. We do not believe that this approach will be as effective as antibodies against dimers for two important reasons. Firstly, it has been established that dimers are toxic species but there is no clear evidence that monomers have in themselves a pathologic role. Secondly, monomers may well have a functional role and so the use of antibodies against them may have detrimental effects. However, this approach could prevent the formation of dimers and hence reduce plaque formation and so it is of interest to use the model to make predictions about the outcomes of such a procedure and to compare the efficiency of preventing plaque formation using antibodies against monomers and dimers. We used the same method as for antibodies against dimers except we replaced antiAbDim with a molecular species named antiAbMon which we assumed could bind to monomers and so prevent the monomers from forming dimers or being sequestered into plaques. Interestingly our model predicted that using antibodies against monomers requires that the addition of antibodies is two orders of magnitude higher than for dimers in order to reduce the level of plaques to those shown in Figure 8. This is because Aβ monomers are continually being produced and so a large number of antibodies are required to prevent any dimerisation taking place and to prevent the sequestering of monomers into aggregates. Our model also predicted that if the addition of antibodies against monomers was carried out at age 70 or 80, there was an initial significant decrease in the number of cells associated with plaques which was due to increased disaggregation. However, at any age of administration the maximal effect was only temporary as antibodies became bound to monomers depleting the available pool (Figure 8B).

### Inhibition of Aβ production

#### Model predicts that inhibition of Aβ production is less effective than targeting dimers especially for interventions at early ages

Interventions to decrease Aβ production, such as secretase inhibitors, are also being tested [39]. Therefore it is also of interest to use our model to mimic the action of such an intervention by decreasing the rate of Aβ production. As in the antibody simulations, we simulated the intervention starting at different ages. The model predicts that a 25% reduction in the production rate slows down the rate at which plaques form but does not completely prevent the increase in plaque formation (Figure 8C). In particular, an early intervention at age one year greatly reduces the plaque burden but does not completely prevent it as was seen in the immunization simulations (compare pink curves in Figures 8A and 8C). Therefore our model predicts that interventions to reduce Aβ production may not be sufficient to prevent plaque formation unless administered at high doses and for prolonged time periods.

### Model 3 plaques grow by addition of dimers

#### The hypothesis that plaques grow by addition of dimers is not supported by our model

**Figure 9:**
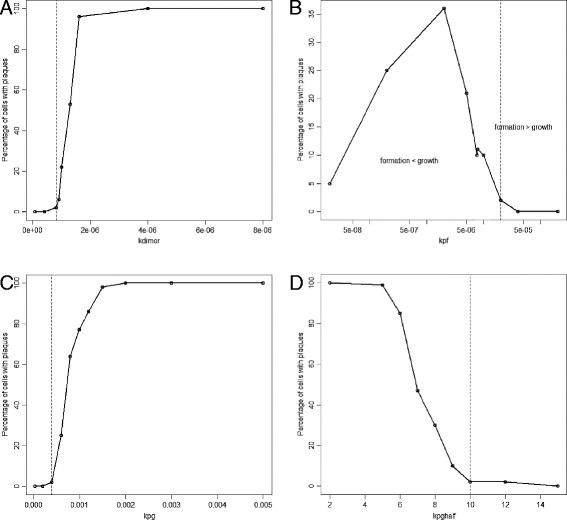
**Simulation results showing sensitivity of parameters for Model 3. A***k*_*dimer*_, **B***k*_*pf*_, **C***k*_*pg*_, **D***k*_*pghalf*_. Each point shows the percentage number of cells associated with plaques at age 100 years from 100 simulations. Dashed black lines indicate the default value of the parameter. Red dashed line in B indicates where the rate of plaque formation is approximately equal to the rate of plaque growth.

Model 2 was modified so that plaques grow by the addition of dimers instead of monomers and so that disaggregation of plaques releases dimers. As for Model 2, we chose a parameter set such that very few plaques form under normal Aβ clearance but that at least 25% of cells are associated with plaques by age 100. The parameters in this model are much more sensitive to perturbation by small stochastic effects than in Model 2, and the model predicts that varying all the parameters apart from *k*_*pf*_, produces three distinct regions in the plots of percentage cells associated with plaques versus parameter value (Figure 9). These correspond to values where firstly no cells develop association with plaques; secondly all cells develop association with plaques; and thirdly, an intermediate situation exists. For the parameter *k*_*dimer*_, the intermediate range is very small, showing that this is a very sensitive parameter in this model. When the parameter *k*_*pf*_ is varied, a very different shape to the plot emerges. Initially increasing *k*_*pf*_ led to an increase in the percentage of cells associated with plaques but after a maximum of 35% was attained, any further increases in *k*_*pf*_ led to a reduction in plaque levels. This behaviour may seem rather strange but is the result of both plaque formation and plaque growth depending on the availability of Aβ dimers in this model. If the rate of plaque formation exceeds the rate of plaque growth, then plaques keep forming, disaggregating and reforming but they cannot grow and cannot reach the size at which plaque growth becomes irreversible (dashed red line in Figure 9B indicates value of *k*_*pf*_ where rate of plaque formation is approximately equal to rate of plaque growth). Most of the parameters are very sensitive to small changes with only small regions where just a proportion of cells are associated with plaques as opposed to all or none. This suggests that Model 3 is less likely to be biologically plausible than Model 2. Therefore, our models provide strong support for the current consensus that plaque growth is mediated by monomers rather than dimers.

## Discussion

We have shown how a modelling approach can be used to address questions concerning the aggregation of amyloid-beta in Alzheimer’s disease. For many readers, this approach may be unfamiliar and some may consider it premature and incomplete. However, we would like to point out that this is exactly why this paper is both timely and valuable. We developed a stochastic model which could be simulated over long time periods to investigate the effects of Aβ turnover on the accumulation of plaques with age. The model showed that dimers play an essential role in seeding (or starting) the aggregation process and that targeting Aβ dimers rather than monomers is likely to be the best approach for future interventions. The motivation for the model was to examine in detail the basic steps in the process of amyloid aggregation and to use the model to make predictions about the effects of proposed interventions. There are many different hypotheses concerning the aggregation process in AD and great efforts are being made to find effective interventions. Modelling is a useful tool for testing ideas and examining the effects of proposed interventions being able to make predictions which can be tested experimentally. For example, our model predicted that very early interventions could prevent the formation of aggregates at later ages. This could be tested experimentally in an animal model of AD and/or an animal model of trisomy 21.

We kept the model as simple as possible and so all the processes that are included in the model are essential to reproduce the results shown here. Stochastic simulation was necessary in order to investigate the cellular variability in plaque formation. Each simulation run represents the outcome for one brain cell and we carried out 500 simulations for each parameter set. The simulation output for 500 cells usually contain cells that do not develop any association with plaques over a 100-year period (depending on the parameter set) and in cells where plaques form, the time of formation is very variable. Our models predict that dimerisation of Aβ, which is the first step in the aggregation process, has a large effect on both the timing of plaque formation and the probability of a plaque forming.

We used the model to examine the effects of immunization against dimers by simulating the addition of antibodies at different ages followed by continued provision of antibodies. This more closely resembles passive immunization with continual provision of antibodies. Ideally, a model of active immunization would be preferable as this procedure would act much better clinically. However, active immunization is a complicated process and would require a more complex model than we have attempted here. Although we used a simple method to mimic the process, the findings are still valuable and show that it would be worthwhile to modify the model for active immunization. An important finding from the simulations is that the use of antibodies against dimers could greatly reduce plaque burden and that they would be much more beneficial if used in mid-life rather than in old age, but that benefit is provided even if administered at later ages provided the patient did not already have a high burden of plaques. An exciting possibility is the development of a vaccine for babies with Down’s syndrome to prevent the early onset of AD. The current model has focussed on the role of amyloid plaques in AD although the actual role of plaques in the disease process is still not clear. On the one hand some studies have shown a lack of correlation between plaque burden and the duration or severity of disease [40,41]. Conversely, another study showed an increase in plaques in the neocortex which correlated with severity of dementia and the authors concluded that plaques are not a consistent feature of ageing [42]. Plaques are found in regions of the brain that are affected in AD and they are usually associated with neuronal processes causing synaptic loss and so may disrupt communication between neurons. However, the exact role of plaques in synaptic loss is still not clear [43]. Support for the role of plaques in AD comes from post-mortem studies of brain specimens from participants in the Phase 2a active immunization trial that there was a significant reduction in amyloid pathology which correlated with an improvement in neurite abnormalities in the hippocampus and an amelioration of tau pathology [44]. Our model does not currently include any pathogenic role for plaques, rather we just used them as a measured outcome. A future development of the model would be to extend it to include detail of synaptic function and include possible mechanisms for how this could be disrupted by plaques.

Our models investigated only the role of monomers and dimers in the aggregation process but could be extended to include further steps such as larger oligomers and protofibrils. This could be achieved by adding further species to the model and replacing the reaction of two dimers forming a plaque with a series of reactions. This would allow the model to be used to examine the effects of interventions on soluble oligomers. Since soluble oligomers may be more toxic than the fibrillar form of amyloid, this would be very desirable in order to use the model to test interventions prior to clinical trials. However, it should be noted that this will make the model more complex and slower to simulate, and we would need further information on how the different amyloid components adversely affect cellular processes. As the model is encoded in SBML, any future developments will be straightforward. Although we focus here on the role of amyloid in AD, we have previously modelled the aggregation of both Aβ and tau in a more complex model [29]. However, the previous model could only be used to mimic cellular models of aggregation over time periods of days rather than years due to the greatly increased computational load. A potential compromise in future studies might be to take some of the key steps involved in tau aggregation and add them to the model of Aβ turnover and aggregation. We could also extend the model to include microglia and then use the model to examine the effect of immunotherapy against amyloid plaques. Another important extension to the model would be to add apolipoprotein E (APOE) which exists as three different isoforms (APOE2, APOE3 and APOE4) with APOE4 being known to have a pathogenic role in AD [45]. A particularly relevant role of APOE in AD is that the APOE-lipoprotein binds to Aβ and is involved in Aβ clearance. However APOE4 is less efficient than the other isoforms in this role, which could contribute to its pathogenicity [46]. Finally we could extend the model to examine the interaction between protein aggregation and mitochondrial dysfunction in AD. For example we could add the interaction between APP, Aβ and mitochondria (Figure 1) as there is evidence that the accumulation of full-length APP and Aβ in the mitochondrial compartment has a causative role in impairing mitochondrial physiological functions [47]. We could also use the model to examine other possible therapies such as promoting degradation of Aβ by administering agents that enhance the activity of Aβ-degrading enzymes [28]. Our model predicts that lower levels of neprilysin leads to an increase in plaque formation and so our model supports that the use of such therapies could be beneficial. We could use our model to test this by simulating the addition of extra neprilysin at different time-points. This might require inserting more detail of the mechanisms involved in the decline in neprilysin activity with age. This approach could also be relevant for other diseases such as cerebral amyloid angiopathy since it has recently been shown that neprilysin protects cerebrovascular smooth muscle cells against Aβ induced degeneration [48].

## Conclusions

Progressive ageing of populations around the world, has been accompanied by alarming growth in the numbers of patients diagnosed with AD and other dementia-like illnesses. The large number of studies showing a correlation between synaptotoxic amyloid species, synapse loss and cognitive deterioration which clinically characterises AD patients, strongly suggests that targeting abnormal levels of Aβ in the brain might provide a potentially powerful preventative strategy. Nevertheless, there remain important questions about how such interventions might act upon the normal mechanisms for formation and degradation of protein aggregates within the brain. It is important to understand the kinetics of these processes, which are significantly influenced by the actions of chance at the levels of the underlying molecular interactions. By employing stochastic mathematical modelling, the present study served to provide evidence for the validity of following such an approach. Moreover, Aß dimer formation was highlighted as a particularly important contributing molecular element underlying AD pathology, in agreement with recently published experimental studies. In conclusion, the study served to provide better insight into the kinetic processes underlying eventual fibril/plaque formation, which might help pinpoint where and at which time-point in such processes is the most therapeutically promising targets for therapeutic intervention.

## Methods

We began by building a model of Aβ turnover, which includes reactions for Aβ production and degradation but does not consider the aggregation process. The aim of this model, which we refer to as Model 1, was to see the effect of decreased clearance on levels of Aβ over time. We then extended this model to include details of Aβ aggregation (Model 2) to examine the effects of decreased clearance of Aβ on the aggregation process. We also examined a variation of Model 2 in which a different assumption was made concerning the growth of plaques. Details of all three models are given below.

### Model 1: Aβ production and degradation

This model contained just one molecular species, Abeta, and two reactions, synthesis and degradation:

(1) $$Source\;\overset{k_{\mathrm{prod}}}{\to }\;Abeta$$

(2) $$Abeta\;\overset{k_{\deg}}{\to }\;Sink$$

We used mass action kinetics which means that the rate of a reaction is directly proportional to the number of molecules (note that we use stochastic simulation; in a deterministic model number of molecules would be replaced by concentration) of each reactant raised to the power of its stoichiometry. In the first reaction equation, Source is a constant dummy variable and so this is a zeroth-order reaction with a constant production rate and *k*_*prod*_ is the hazard of this reaction. The second reaction equation is of first-order and the hazard of this reaction is *k*_*deg*_ Abeta. Details of how the model was coded and simulated are given later in the Methods section under the heading “Tools used for model construction and simulation”.

In order to simulate the model, we need to choose values for the parameters and the initial amount of Abeta. We used the measured Aβ production rate (*k*_*prodAbeta*_ = 1.86e-5 molecules s^-1^) from Mawuenyega and colleagues [22]. The degradation rate depends on the concentration of Aβ in the cell (e.g. *k*_*degAbeta*_ * Abeta). Therefore we need to know the steady state level of Aβ. The concentration of Aβ_42_ was measured in CSF and was found to be 500pg/ml for patients with normal Aβ clearance rates [22] and gives no indication about the level of soluble Aβ_42_ at the cell surface where it is produced. Usually, Aβ is rapidly cleared and therefore we would expect the level of Aβ in the vicinity of the membrane to be low, especially as APP is found throughout the cellular membrane and is not localised to a particular position. Therefore we assume that the local level of Aβ is initially given by Abeta = 1, and assume *k*_*degAbeta*_ = 2.1e-5 s^-1^ for the normal degradation rate and *k*_*degAbeta*_ = 1.5e-5 s^-1^ for the degradation rate in AD. The model was simulated 500 times for a particular set of parameters over a time period of 100 years and the level of Aβ was output to a file (500 time-points were chosen). The results were analysed and plotted in R (Figures 1,2,3,4).

### Model 2: Aβ turnover and aggregation

We extended Model 1 to include the aggregation of Aβ. To keep the extended model as simple as possible we make the following assumptions.

1. Two Aβ monomers may interact to form a dimer.

2. Dimers may disassociate into two monomers.

3. Two dimers may interact to form a small aggregate.

4. Aggregates may grow by the addition of monomers only (although we also consider growth by dimers in Model 3).

5. Aggregates may disaggregate to release a monomer.

6. When aggregates reach a certain threshold size, they continue to grow until they reach a maximum size. Once the aggregate has passed the threshold size we consider it to be a plaque.

7. Since the actual size of the threshold is not fixed, i.e. it varies in a stochastic fashion, we do not have separate species for small aggregates and plaques and use the name AbP for all aggregates. Therefore, in our model output analysis we assume that if AbP is greater than a certain level then it is counted as a plaque. We chose 30 for this threshold size as plaques of this size, or larger, rarely completely disaggregated.

## Rate laws and parameter values

### Parameters for Aβ turnover

We used the same values for Aβ production and degradation as in Model 1. However, once we added the aggregation steps, we found that the model predicted plaques could occur at early ages when we set the degradation rate at the AD level. Originally we assumed that Aβ clearance was impaired throughout life. However, with this assumption it was not possible to find a parameter set where levels of plaques are very low early in life but increase only after the age of 60 years (Figure 5B). There are 3 possible ways that we could modify the model to provide more realistic output. Firstly, the parameters which affect lag time could be adjusted. Secondly, further steps could be added to the aggregation process such as formation of trimers, oligomers and protofibrils, in order to increase the lag time. Finally, we assumed that the degradation rate for AD is lower than normal throughout life but it is more likely that the rate declines with age. There are no parameters in the model which affect only lag time (discussed in further detail in the Results section). We did not wish to add further complexity to the model, so did not consider the second possibility. The assumption of a constant low degradation rate is probably not correct since if this was the case, then we would expect to see cases of sporadic AD at earlier ages. Therefore we chose to amend the model so that the degradation rate declines with age. We added one of the Aβ degrading enzymes to the model, namely neprilysin, since it is known that its level decreases with age. We assumed that the Aβ degradation rate depended on the level of neprilysin and that the level of neprilysin declined with age in AD. Therefore we needed an additional parameter, *k*_*degNep*_, for the degradation rate of neprilysin. We chose the value for this so that neprilysin values declined by about 30% by age 60 which resulted in Aβ degradation rates similar to those observed in AD patients at late ages.

### Parameters for Aβ aggregation

**Figure 10:**
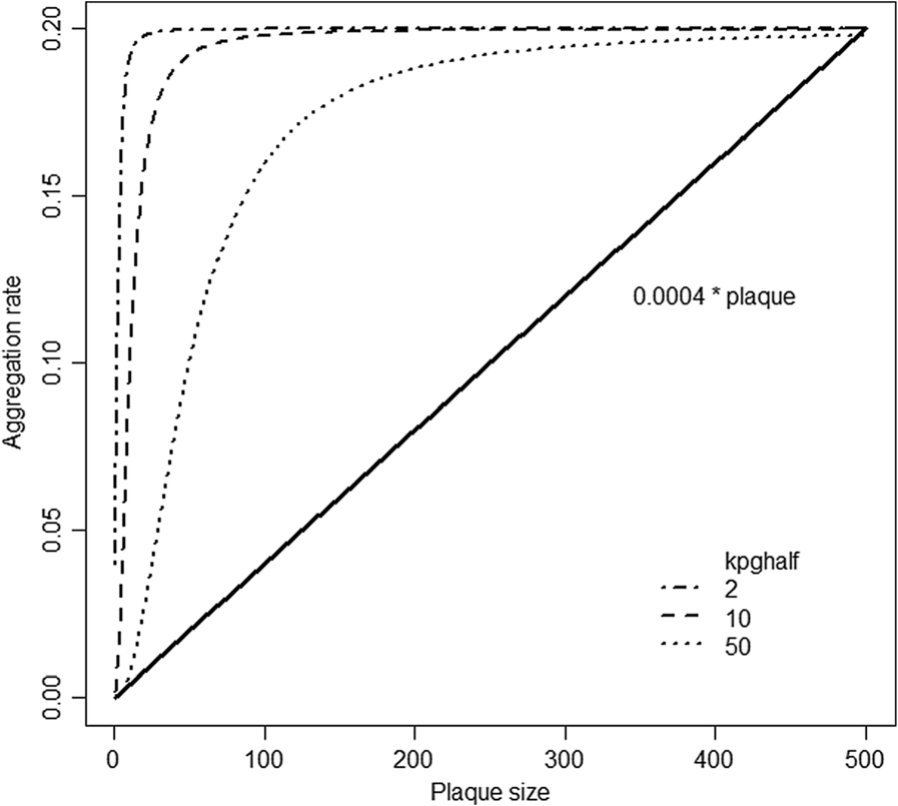
**Rate of plaque growth versus plaque size for different rate laws.** The continuous line shows a mass action rate law with parameter 0.0004 and shows that the aggregation rate increases linearly with plaque size. The dashed and dotted lines show a rate law with Hill kinetics and the effect of changing the parameter which represents the level of plaques where the rate is half the maximal value.

We needed to include an additional five reactions and six parameters to model the kinetics of Aβ aggregation. These are shown in Tables 2 and 3 and described in more detail below. *In vitro* experiments show that once plaques start to form, they grow rapidly (within about 24 hours) and then stop growing, i.e. they reach a maximum size. Since Aβ is continually produced, this observation suggests that degradation of plaques takes place so that the process is not irreversible and that at a certain size disaggregation balances plaque growth. It has been shown that plaques have a porous structure and so the rate of disaggregation is likely to be proportional to the volume of the plaque [49]. Therefore, we use first order mass action kinetics for disaggregation and assume that the rate depends only on the size of the aggregate. The rate of aggregate growth depends on the availability of Aβ dimers (AbDim) and/or monomers (Abeta). The rate law must also contain the species AbP (aggregates) so that aggregates cannot grow unless AbP is greater than zero (if AbP = 0, then the rate of aggregate growth must also equal zero). However, the rate of growth cannot depend linearly on aggregate size, otherwise, the rate of growth would continue to increase and plaques would keep growing (Figure 10, black line). Instead we assume that initially the rate of plaque growth increases with plaque size but that it reaches a maximum rate (*k*_*pg*_) at a particular plaque size which is determined by the parameter *k*_*pghalf*_ (Figure 10, curved lines). This can be achieved by using a Hill function so that the rate law for plaque growth is given by;

(3) $$\text{Plaque growth rate}=k_{\mathrm{pg}}Abeta\;\left(\frac{AbP^{2}}{k_{\mathrm{pghalf}}{}^{2}+AbP^{2}}\right)$$

**Table 3:** List of reactions for Model 2

**Reaction id**	**Reactants**	**Products**	**Kinetic rate law**
AbetaProduction	Source	Abeta	*k*_*prod*_
AbetaDegradation	Abeta	Sink	*k*_*deg*_ Abeta (Normal rate)
			*k*_*deg*_ Abeta * Nep (AD rate)
Dimerisation	2Abeta	AbDim	*k*_*dimer*_ Abeta*(Abeta-1)*0.5
Dedimerisation	AbDim	2Abeta	*k*_*dedimer*_ AbDim
PlaqueFormation	AbDim	AbP	*k*_*pf*_ AbDim*(AbDim-1)*0.5
PlaqueGrowth	Abeta, AbP	2AbP	*k*_*pg*_ Abeta*(AbP^2^/(*k*_*pghalf*_^2^ + AbP^2^))
Disaggregation	AbP	Abeta	*k*_*disagg*_ AbP
NeprilysinDegradation	Nep	Sink	*k*_degNep_*Nep

We classify aggregates containing more than 30 monomers as plaques (i.e. AbP >30), as our model output shows that once aggregates reach this size, they are unlikely to disaggregate completely but continue to grow in size. Therefore, our use of the term “plaque” incorporates all aggregates of Aβ which have exceeded a critical threshold size and have a high probability of forming the amyloid deposits which are termed “plaques” in the disease state.

The rate of fibril formation and fibril growth has been measured experimentally and it has been reported that fibrils grow at a rate of 0.5 monomers per minute and that new fibril nucleation takes place approximately once in 5 days per micelle [13,50]. We chose values for *k*_*pf*_ and *k*_*pg*_ based on these measurements. We do not have data for the rates of dimerisation, disassociation of dimers or disaggregation of plaques and so we used a global parameter scan in order to find parameter sets which predict very low levels of plaques before the age of 50 years and then a gradual increase in the number of plaques up to age 100 years (the maximum age used in our simulations). In addition, we assumed that the parameters need to satisfy the condition that levels of plaques do not exceed 10% by age 100 years when Aβ clearance is normal, but that for impaired Aβ clearance, levels exceed 25% by the age of 100 calendar years. We do not have data on the percentage of cells associated with plaques in normal ageing and AD, but these values seemed reasonable. In future, these values may be measurable and then it would be possible to fit parameter sets to clinical data.

**Figure 11:**
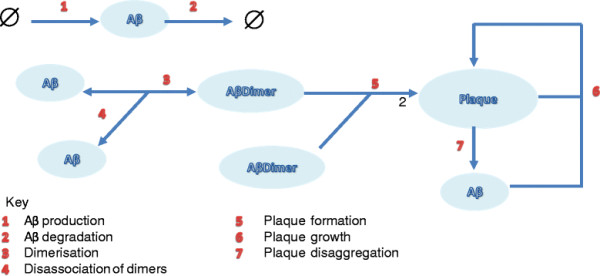
Diagram of the network for Model 2.

Since we need to use stochastic simulation, it is not practical to do a full global parameter scan. Instead we used Latin hypercube sampling to generate 100 parameter sets. We assumed that the values of *k*_*prod*_ and *k*_*deg*_ are fixed, and constrained *k*_*pf*_ and *k*_*pg*_ to be within half and twice the values given by experimental data [13]. The other parameter values were allowed to vary by two orders of magnitude. Initially we did 10 simulations for each parameter set for the normal Aβ clearance rate. We eliminated any sets where more than 30% of the simulated cells produced plaques by age 100. We chose 30% rather than 10% since we initially only carried out 10 simulations for each parameter set to save computer time and did not want to eliminate sets which by chance had a high proportion of plaques. We then carried out 100 simulations for all the retained sets (64 sets) for the impaired Aβ clearance rate (*k*_*deg*_ declines with age) and eliminated any sets which either produced less than 10% of cells associated with plaques by age 100 or produced too many plaques (more than 15%) before the age of 50 years. This resulted in 10 possible sets. We did further simulation runs for all these sets and finally chose the set which gave the best difference between plaque levels for normal and decreased clearance of Aβ. Details of the parameter sets used in this analysis are given in Additional file 1. Full details of the reactions and parameters are given in Tables 2,3, and 4. A diagram of the network is shown in Figure 11.

**Table 4:** List of species for Model 2

**Species id**	**Description**	**Initial value**
Abeta	Aβ monomer	0
AbDim	Aβ dimer	0
AbP	Plaque	0
Nep	Neprilysin	1000

### Model 3: Aβ turnover and aggregation with plaque growth via dimers

Although the current consensus holds that plaque growth is via monomers, it cannot be ruled out that dimers provide the building blocks for such pathological aggregates [51]. To examine this possibility, we changed the reactions for plaque growth and disaggregation. This model was fitted with different parameter values and did not receive such extensive analysis as shown in Model 2. The parameters are much more sensitive in this model. Apart from *k*_*pf*_ all the parameters can take values that result in no cells associated with plaques, all cells associated with plaques or an intermediate level of plaques (Figure 9).

### Tools used for model construction and simulation

The models are encoded in the Systems Biology Markup Language (SBML), a computer-readable format for network models [52]. The tool used to create the SBML code was SBML short-hand which uses a Python script to convert a short-hand version of SBML into the full code [53]. The code is available from the Biomodels database (Biomodels ID: MODEL1202290000)[54,55] and is also provided in Additional file 2. The SBML model can be imported in any software tool which has the facility for stochastic simulation. The stochastic simulations were carried out using the gillespie2 code (available from the SBML website [56]), which is based on the Gillespie algorithm [57]. Briefly, this algorithm uses random numbers to simulate the time to the next event and to pick the reaction that occurs at this time according to the reaction hazard. The number of molecules of each species is updated according to the reaction which occurred. The procedure is repeated until the simulated time to the next event exceeds the maximum time of the simulation. The model results were analysed and plotted using the R statistical package.

### Statistical analyses

The slopes of the regression lines in Figure 4 were tested for statistical significance using a student’s unpaired t-test. The correlation between two variables was calculated using Pearson’s r statistic. The 95% confidence interval error bars for the percentage of cells associated with plaques were calculated using ±1.96 x s.e., where s.e. = √(p(100-p)/N), p is the percentage and N is the number of simulated cells. All calculations were carried out in the R statistical package.

## Abbreviations

AD, Alzheimer’s disease; APP, Amyloid precursor protein; APOE, Apolipoprotein E; CSF, Cerebrospinal fluid; IDE, Insulin-degrading enzyme; LTP, Long-term potentiation; SBML, Systems biology markup language; SDS, Sodium dodecyl sulphate.

## Competing interests

The authors declare that they have no competing interests.

## Supplementary Material

Additional file 1 This file contains details of parameters used in the global parameter scan for Model 2 showing how the best parameter set was chosen. Click here for file

Additional file 2 This file contains the SBML code for Model 2 with Aβ degradation rate decreasing with time. Click here for file
